# Draft genomes of *Aspergillus nomiae* and *Aspergillus tamarii* isolates from human eye infections

**DOI:** 10.1128/MRA.00391-23

**Published:** 2023-07-17

**Authors:** E. Anne Hatmaker, Jonathan E. Schmitz, Antonis Rokas

**Affiliations:** 1 Department of Biological Sciences, Vanderbilt University, Nashville, Tennessee, USA; 2 Vanderbilt Evolutionary Studies Initiative, Vanderbilt University, Nashville, Tennessee, USA; 3 Department of Pathology, Microbiology, and Immunology, Vanderbilt University Medical Center, Nashville, Tennessee, USA; 4 Vanderbilt Institute for Infection, Immunology, and Inflammation, Nashville, Tennessee, USA; University of California, Riverside, Riverside, California, USA

**Keywords:** fungal keratitis, *Aspergillus*, *Aspergillus flavus*, pathogenicity, clinical isolate

## Abstract

Fungal species within the genus *Aspergillus* are an important cause of human diseases, including aspergillosis and keratitis. We present draft genomes of *Aspergillus tamarii* and *A. nomiae* isolated from keratitis patients in a U.S. tertiary care hospital.

## Announcement

Fungal keratitis (FK) is a serious infection of the eye cornea that can lead to blindness (1). Individuals are typically infected through small wounds on the eye’s surface; the disease primarily afflicts outdoor workers and contact lens wearers (2, 3). At least a million new FK cases are reported annually worldwide (4). Fungi within section *Flavi* (a taxonomic rank between genus and species) of the genus *Aspergillus* are a leading cause of FK in humans (5). The section includes the opportunistic pathogen *Aspergillus flavus*, as well as *Aspergillus nomiae* (formerly *Aspergillus nomius*) and *Aspergillus tamarii* (6).

We obtained two FK isolates from the Vanderbilt University Medical Center (VUMC) clinical microbial biorepository microVU, under appropriate Institutional Review Board review (protocol #210613). Samples were collected during routine clinical care at VUMC and isolated from patients prior to 2020. Using morphological characteristics, both samples were identified as members of *Aspergillus* section *Flavi*. Isolates were received from the culture collection on blood agar slants and subsequently cultured on potato dextrose agar and incubated at 37°C for 7 days. Mycelia were then collected in a 1.5-mL Eppendorf tube and homogenized with silica beads (ThermoFisher Scientific, Waltham, MA, USA). DNA was extracted using the Qiagen Plant DNEasy kit according to the manufacturer’s protocols. Libraries were prepared using the TruSeq DNA PCR-free kit (Illumina, San Diego, CA, USA). Paired-end sequencing of 150 bp reads was performed at the Vanderbilt Technologies for Advanced Genomics facility in Nashville, TN, USA using the Illumina NovaSeq 6000 platform (Table 1).

**TABLE 1:** Assembly and genome assessment statistics for the two clinical isolates, *A. tamarii* VU91 and *A. nomiae* VU104

		*Aspergillus tamarii* VU91	*Aspergillus nomiae* VU104
Sequencing results	Raw reads (paired)	48,794,587	39,927,203
	Trimmed reads (paired)	36,286,541	31,033,446
BUSCO analysis	Present (% out of 4,191)	99.5	99.4
	Complete	4,168	4,166
	Complete, single copy	4,152	4,151
	Complete, duplicated	16	15
	Fragmented	3	3
	Missing	20	20
Nuclear genome statistics	No. scaffolds	307	839
	Coverage	142×	124×
	N50	757,988	621,240
	Size (Mbp)	38.07	37.49
	GenBank accession	JARLUE000000000	JARLUD000000000
Mitochondrial genome statistics	No. scaffolds	1	1
	No. mapped reads	598,257 (1.75%)	1,134,321 (3.87%)
	Coverage	3,022×	5,607×
	Size (bp)	29,691	30,342
	No. predicted proteins	14	14
	GenBank accession	OQ916342	OQ916343

Sequences were filtered for quality, and vectors were trimmed using Trimmomatic v0.39. Genomes were assembled using SPAdes v3.15.3 (7), with the inclusion of the “--careful” flag and specific k-mer settings (21, 33, 55, 77, 99, and 127). Scaffolds shorter than 500 bp were removed from the assemblies. Mitochondrial genomes for both isolates were single scaffolds within the whole-genome SPAdes assemblies and were annotated using GeSeq v2.03 (8). Average nucleotide identities (ANI) for both genomes were calculated using fastANI v0.1.3 (9) within KBase v2.7.2 (10). Genome completeness was evaluated using the “eurotiales_odb10” database within BUSCO v4.0.4 (11) (Table 1). The clinical isolates were identified as *A. tamarii* and *A. nomiae* through ANI comparisons to genomes of multiple *Flavi* species from NCBI. VU91 shared 99.3% similarity with *A. tamarii* CBS117626 (GenBank: GCA_009193485) and 88.2% with *A. flavus* NRRL3357 (GenBank: GCA_009017415.1). VU104 shared 99.2% similarity with *A. nomiae* NRRL13137 (GenBank: GCA_001204775.2) and 87.4% with NRRL3357.

Both *A. tamarii* and *A. nomiae* have been previously isolated from FK patients, but they are rare compared to infections caused by other *Aspergillus* section *Flavi* species such as *A. flavus (*12, 13) . Despite the differences in prevalence among patients, recent work showed that the virulence of an *A. nomiae* strain was similar to that of some *A. flavus* strains in an invertebrate model of fungal disease (14). Genomic information from clinical isolates of section *Flavi* species is currently lacking in public databases, despite the impact of these species on human health. The two draft genomes presented here enable comparisons of clinical and environmental *Aspergillus* isolates, providing valuable data for future studies on their mechanisms of pathogenicity and virulence.

## Data Availability

The complete assemblies and paired-end reads of both genomes are available from NCBI’s GenBank and SRA, respectively. All data associated with this project can be found under NCBI BioProject accession number PRJNA945023. Aspergillus tamarii VU91 data is associated with BioSample SAMN33771164. The Whole Genome Shotgun sequencing project has been deposited in GenBank under accession JARLUE000000000 and the version described in this paper is version JARLUE010000000. Aspergillus nomiae VU104 data is associated with BioSample SAMN33771165. The Whole Genome Shotgun sequencing project has been deposited in GenBank under accession JARLUD000000000 and the version described in this paper is version JARLUD010000000.
